# Flight Phases in the Song of Skylarks: Impact on Acoustic Parameters and Coding Strategy

**DOI:** 10.1371/journal.pone.0072768

**Published:** 2013-08-15

**Authors:** Juliette Linossier, Fanny Rybak, Thierry Aubin, Nicole Geberzahn

**Affiliations:** 1 Université Paris-Sud, Centre de Neurosciences Paris-Sud, UMR 8195, Orsay, France; 2 Centre National de la Recherche Scientifique, Orsay, France; Claremont Colleges, United States of America

## Abstract

Skylarks inhabit open fields and perform an aerial song display which serves as a territorial signal. The particularly long and elaborate structure of this song flight raises questions about the impact of physical and energetic constraints acting on a communication signal. Song produced during the three distinct phases of the flight - ascending, level and descending phase could be subject to different constraints, serve different functions and encode different types of information. We compared song parameters during the ascending and the level phases. We found that the structure of the song varied with the phase of the flight. In particular, song had a higher tempo when skylarks were ascending which might be related to higher oxygen and energetic demands. We also explored which phase of the song flight might encode individuality. Earlier studies reported that skylarks reduced their territorial response to established neighbours if the neighbour song was broadcasted from the correct adjacent boundary, but reacted aggressively if the neighbour songs were broadcasted from an incorrect boundary (mimicking a displaced neighbour). Such differential response provides some evidence for individual recognition. Here, we exposed subjects to playback stimuli of neighbour song in which we had replaced either the song produced during the level or the ascending phase by the relevant song of the neighbour from the incorrect border. Singing response was higher towards stimuli in which the ‘level phase song’ was replaced, indicating that skylarks could be able to recognise their neighbours based on song of this phase. Thus, individuality seems to be primarily coded in the level phase of the flight song.

## Introduction

Bird song transmits different types of information such as species identity, group label, individual identity and motivational state [1], that are often encoded in the song at different levels. For instance, species identity can be based on acoustic parameters such as rhythm (e.g. indigo bunting, *Passerina cyanea*, [2], skylark, *Alauda arvensis*, [3]), individual and group identity may be encoded through the composition of syllable sequences (skylark, [4]) and aggressive motivation can be signalled by amplitude parameters (e.g. song sparrow, *Melospiza melodia*, [5]), frequency parameters (e.g. African black coucals, *Centropus grillii* [6], [7]) or song density (ratio of all syllable durations by overall duration of a song, skylarks, [8]). One single information can also be coded at several levels of the song structure, as for example in the canary *Serinus canaria*, where both acoustic features and repertoire composition provide individual information [9].

Despite variations in coding parameters, and on more proximate terms, variation in the song structure produced by different species also reflects differences in the structure of the sound producing organ and in its nervous and muscular control. The production of a song requires the coordination of three main motor systems (respiration, syrinx and vocal tract, [10]).

There are several constraints imposed on the mechanisms of sound production, such as the limit of volume of air inhaled/exhaled, anatomical and physiological limitations (body size, syrinx size, metabolic status, performance of the respiratory muscles,) and environmental limits (air pressure, temperature,) (Table 1 in [11]).Birdsong is typically produced during pulses of expiratory air pressure that alternate with short inspirations (minibreaths, e.g. [12]). The temporal pattern of syllable production is often determined by the respiratory rate [10]. Thus, the needs in oxygen and constrains exerted on the respiratory system are likely to impact the production of song and in particular its acoustic structure. Such links between sound production, breathing and muscular activity are likely to be particularly tight in species that produce song and fly at the same time. Given that during flight oxygen consumption increases (evening grosbeak, *Coccothraustes vespertinus*,[13]) breathing rate might be increased and accordingly the acoustic structure of song produced by such species during flight may reflect the physical and metabolic constraints exerted by the flight. Likewise, acoustic parameters of the song could vary if oxygen demands change during different phases of a flight song.

**Table 1:** Measures of response recorded during playback experiments.

Measured variable
Total duration of movements (s)
Duration of movements between 10 and 5 m from the loudspeaker (s)
Duration of movements between 5 and 0 m from the loudspeaker (s)
Latency to the first movement (s)
Latency to approach at less than 10 m from the loudspeaker (s)
Latency to approach at less than 5 m from the loudspeaker (s)
Time spent between 10 and 5 m from the loudspeaker (s)
Time spent between 5 and 0 m from the loudspeaker (s)
Latency before the first song (s)
Duration of songs (s)
Total number of calls

In general, flight song occurs in species living in open habitats, as pipits (tree pipit, *Anthus trivialis*, [14]), larks (skylarks, [15]) and sparrows (swamp sparrows, *Melospiza georgiana*, [16]), and its duration varies between species, from a few seconds as in the tree pipit [14] to few minutes as in the common yellowthroat, *Geothlypis trichas*, [17], to up to 35 min as in the skylark [18]. The case of skylark is particularly interesting because males sing continuously a particularly long and complex song (up to 700 different syllables in the repertoire of an individual, [4]) during flight. This aerial display is divided in three phases: a short ascending phase with flapping flight, a level phase when males alternate flapping and gliding to circle above the territory and a short descending phase when males drop by a semi-gliding flight and plunge to the ground. We here focused only on the ascending and the level phases as the descending phase is often very brief, and birds may even stop singing during this phase (personal observation). We assume that the ascending phase causes higher energetic demands than the level phase because of power requirements for the take-off and because constant wing flapping is required during this phase. These differences could be reflected in acoustic parameters of the song and also could have an impact on the coding strategy. Song information content may thus differ when physical constraints associated to rapid upward flight burden animals with higher energetic costs compared to level flight. According to previous studies, the coding of species identity in skylarks is based on temporal parameters, the rhythm and tempo [3] and the coding of group identity is based on sequences of syllables shared by all the males belonging to the same group constituting "micro-dialects" [4]. In addition, skylarks are able to individually recognize their adjacent neighbours on the basis of their song. This has been demonstrated by playback experiments in which a neighbour's song was broadcasted both from the appropriate and inappropriate territorial border [19]: skylarks reduce their territorial response to established neighbours but react strongly to a neighbour song broadcasted from the opposite side (as such a neighbour might try to expand his territory) providing evidence for individual recognition. However which acoustic code is providing the individual information remains an open question.

The aim of our study was in a first step to investigate whether different constraints on vocal production mechanisms would be reflected in acoustic parameters. We hypothesised that constraints are higher in the ascending phase than in the level phase as ascending and wing flapping probably requires more energy than gliding and circling. Accordingly, different intensities in motor activity in the two phases of flight could lead to differences in temporal and syntactical structure of song. Thus, we examined the potential acoustic differences between the song produced during the ascending phase and the song produced during the level phase at different scales: frequency and temporal parameters of syllables and ordering and versatility of syllables. Furthermore, we conducted a playback experiment in order to test if the two phases provide the same information content regarding the coding of individuality. Songs produced during upward flight phases might be less likely to contain information such as the individual signature. To this end, we observed and quantified the behavioural responses of males subjected to song stimuli simulating a territorial intrusion of a neighbour. Those stimuli were composed of neighbour songs in which we had replaced either the song produced during the level phase or the song produced during the ascending phase by the relevant song of the neighbour from the opposite border. According to previous studies [4], [16], we expected males to react stronger to the part of the song stimuli that they identify as coming from the inappropriate territorial border.

## Materials and Methods

### Ethics Statement

The farmers around the University Paris Sud allowed us to work on their land. No specific permissions were required for this study according to the French legislation. This field study did not involve endangered or protected species. All work conforms to the ABS/ASAB guidelines for the treatment of animals in behavioural research and teaching.

### Study area, subjects and song recordings

This study was carried out during the breeding season, from February to May 2012, in the fields surrounding the University of Paris Sud, France. Twenty males established in 8 different locations (1–3 males per location) were recorded. At a given location, individuals were established in adjoining and stable territories of circa 1 ha. We recorded several complete flight songs per individual between 0800 and 1200 hours Eastern Daylight Time using a Marantz PMD 670 numeric recorder (sampling rate: 44.1 kHz) connected to a Sennheiser ME62 K6 omnidirectional microphone (frequency response: 20 Hz to 20 kHz±1 dB) mounted on a Telinga Universal parabola (diameter: 50 cm). The different phases of flight were assessed by visual observation. To establish a timing of the transition between different flight phases (ascending, level and descending), we made short comments on the recording at each change of phase.

### Song analysis

**Figure 1:**
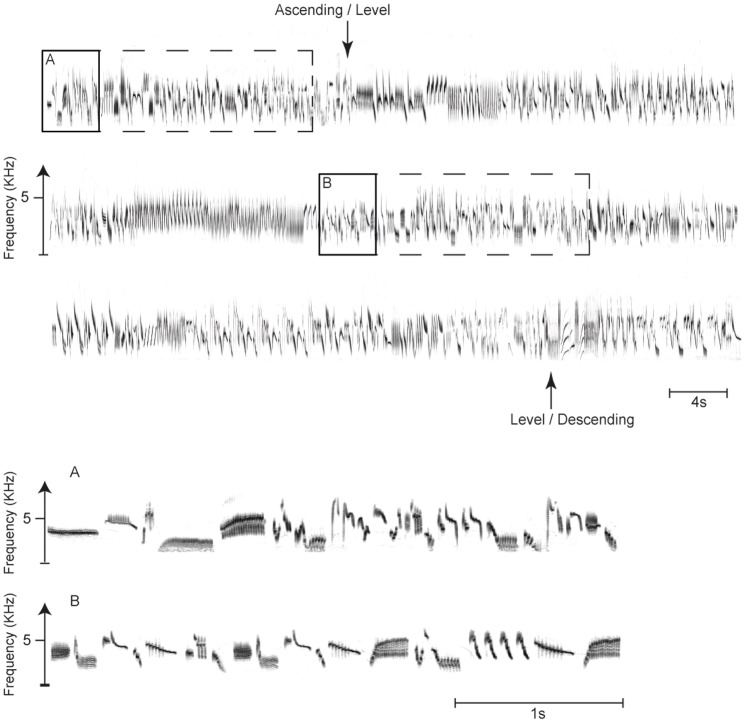
Sound spectrogram of a complete flight song of a male skylark (above) and of two excerpts of the same (below). Dotted line squares highlight 20 s of song produced during the ascending and the level phases as used for acoustic analysis and playback; fine structure of the syllables produced in each phase is shown below (A and B) at higher scale for the song in the continuous line squares. The arrows indicate the transition between the song produced during the ascending, the level and the descending phase.

**Figure 2:**
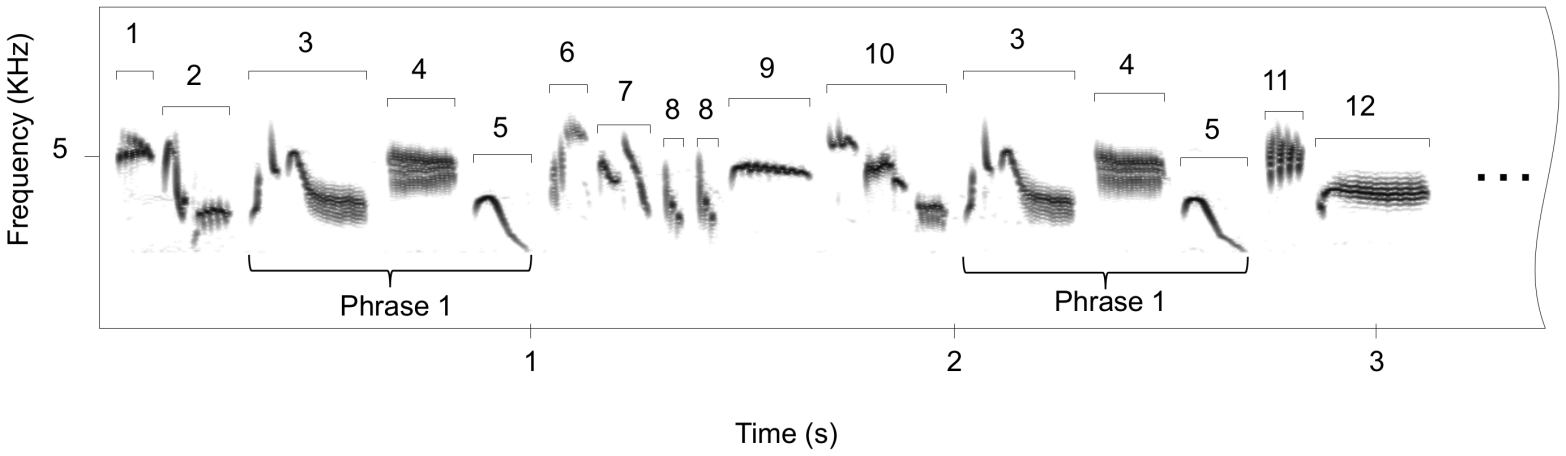
Sound spectrogram of a short part of a flight song of a male skylark. Each syllable is labelled with a number and syllables belonging to the same syllable type have the same number.

For song analyses, we used Avisoft SASLab pro v.5.1.14 software and Praat v.5.3.12. Song files were first high-pass filtered (FFT filtering, cut-off frequency: 1600 Hz) to remove the low-frequency background noise. Then, we selected two songs per individual with the highest signal to noise ratio (N = 40, mean duration: 186±7.1 s). We analysed the first 20 s of song produced during the ascending phase and 20 s of song produced exactly in the middle of the level phase (Fig. 1). Songs were visualized on a sound spectrogram (FFT-length: 1024; Frame: 100%; Hamming window). A syllable was defined as a continuous trace on the sound spectrogram or a group of continuous traces spaced out by less than 25 ms (Fig. 2). Following methods described elsewhere [4] syllables were classified in syllable types according to their overall frequency modulation shapes and labelled on the sound spectrogram with a number. Likewise the same syllable type found in the song of different individuals was labelled with the same number. Then, we examined the sequential organization of syllables using a custom made Matlab program (The MathWorks, Natick, MA, USA; see [4]). To this end, numbers corresponding to the syllable labels were entered into the program in the sequential order of their appearance in the song. By classifying sequences of syllables according to their length and the number of times they were repeated, the program allowed us to detect all sequences that were repeated by the same individual or shared by different individuals. We only considered sequences of at least three different consecutive syllable types repeated by the same individual or shared by different individuals (e.g. Phrase 1 in Fig. 2).

Individuality could be coded by particular syllables or sequences of syllables. Therefore we compared the sharing of syllables and sequences of syllables between neighbours in the song produced during the ascending and the level phases of the flight. We measured such sharing between each pair of individuals within each locality for a subset of subjects (n = 9, 3 birds for each of the 3 localities studied). Coefficients of similarity between repertoires of syllables and coefficients of similarity between repertoires of sequences (sequence length: 3–10 syllables) were calculated as follows: RS = Z/((X+Y)-Z), with X and Y being the total number of syllables or sequences produced by males X and Y, and Z being the number of syllables or sequences shared by males X and Y [20]. RS values range from 0 to 1, with 1 being maximum sharing.

In order to give an account of syllable variety of the song for each flight phase, we calculated the “total versatility” (TotVer), the “syllable type versatility” (SylVer) and the “transition versatility” (TrVer) in sequences of 10 syllables. Syllable type versatility reflects the variety of different syllable types used and ranges from 0 to 10, 10 being the maximum versatility (i.e. the bird sings 10 different syllable types in a sequence of 10 syllables). Transition versatility reflects the variety of transitions used between two different syllables and ranges from 0 to 9, 9 being the maximum versatility (i.e., in a sequence of 10 syllables each syllable differs from the previous one). Total versatility is the product of syllable type versatility and transition versatility and consequently ranges from 0 to 90, with 90 being the maximum versatility (for further details see [21]).

In order to characterize acoustic parameters of each phase of song flight, each syllable was first analysed separately and we then calculated an average for all syllables produced during each phase of the song flight. We measured the following four frequency parameters: the frequency of maximum amplitude (peak frequency, measured on the mean spectrum of the entire syllable), the frequency values at the upper limit of the first (25%), second (50%) and third (75%) quartiles of energy and the bandwidth (difference between maximum frequency and minimum frequency). As temporal parameters, we measured the duration of syllables (duration), the duration of gaps between two successive syllables (gap) and the duration of each syllable plus the successive gap (interval). We then used the temporal parameters to calculate the rhythm (ratio of syllable duration per gap duration) and the tempo (number of syllables per unit of time).

### Statistical analyses used for acoustic parameters

We made all analyses using R v2.13.0, Statistica v6.0 and StatXact v3.1. All means are given ±SEs. We compared acoustic and sequential (versatility) parameters between the ascending and the level phase using GLMs (general linear models). The dependent variables were the song parameters and the independent variables were the phases of flight. Subject identity was included as a random factor. We performed a pDFA [22] to examine which parameters discriminated most strongly the two phases of the flight song by testing for an effect of the predictor variable (phase of flight song) while including individuality as a random factor. We compared RS values calculated for ascending and level phase between males of each locality with two-tailed exact paired permutation tests using the Monte Carlo method [23] [24].

### Playback experiments

#### Subjects and Stimuli

We carried out playback experiments in the middle of the breeding season (April, May) when skylarks exhibit a reduced aggressiveness towards their well-established, and thus trustworthy neighbours (dear-enemy effect, [4]). The playback experiment was conducted on 10 males from 8 locations. These birds were belonging to the sample recorded for the song analysis.

**Figure 3:**
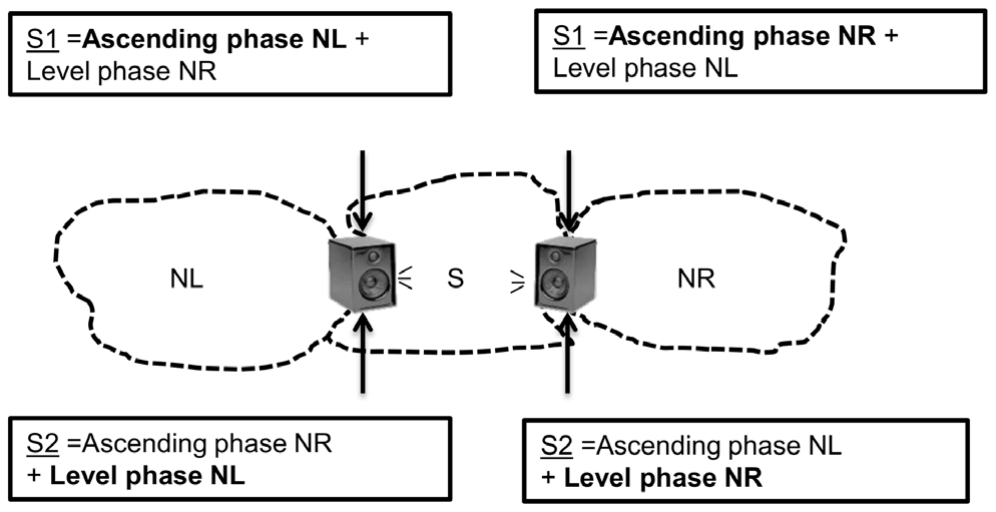
Design of the playback experiment. S is the subject, with its left and right neighbours established respectively in the territories at the left side (NL) and the right side (NR). The composition of each stimulus broadcasted by the loudspeaker placed at either boundary is indicated in the box. Phases of songs broadcasted from the correct side (belonging to the correct neighbour) are in bold. Dotted lines indicate the territorial borders of subjects S, NL and NR. S1 indicate stimuli in which song produced during the ascending phase was broadcasted from the correct and song produced during the level phase from the incorrect side, S2 indicate stimuli in which song produced during the level phase was broadcasted from the correct and song produced during the ascending phase from the incorrect side.

We selected subjects (S) that had two neighbours whose territories were located at opposite boundaries (NL: neighbour left; NR: neighbour right, Fig. 3). We broadcasted 2 stimuli from the boundaries shared with the NL and the NR. To test the respective importance of the song of the two phases of flight in coding individual identity, two types of stimuli were created, in which the song produced during the ascending phase and during the level phase by the two neighbours were combined in two different ways: either the song of the ascending phase was produced by NL and the song of the level phase was produced by NR, or the inverse (Fig. 3). The duration of each stimulus was 80 s in total. We rescaled each song part to root mean square equalized amplitudes using a script implemented in Praat (www.gbeckers.nl/pages/praat_scripts/rms_equalize.praat_script). When played back from the side of NR, stimuli consisting of 40 s of the ascending phase of the NR (the first 20 s of song produced during the ascending phase, broadcasted twice) followed by 40 s of the level phase of the NL (20 second of song produced exactly in the middle of the level phase, broadcasted twice) were named stimuli S1. Likewise, stimuli consisting of the ascending phase of the NL and the level phase of the NR were named stimuli S2. The same design was followed for the playback from the side of NL with S1 corresponding to the ascending phase of NL and the level phase of NR and S2 corresponding to the ascending phase of NR and the level phase of NL (Fig. 3). Thus, S1 were always stimuli with song of the ascending phase broadcasted from the correct boundary and song of the level phase broadcasted from the incorrect opposite boundary. In contrast, S2 were stimuli with song of the level phase broadcasted from the correct boundary and song of the ascending phase broadcasted from the incorrect opposite boundary. This playback design allowed us to test whether individuality is coded rather in the song produced during the ascending or the level phase of the flight. If the ascending phase contains more information on individual identity we expect reduced aggressive response (dear-enemy effect) when the song of the ascending phase is broadcasted from the correct territory border (S1). If, however, the level phase contains more information on individual identity we expect reduced aggressive response if the song of the level phase is broadcasted from the correct territory border (S2).

#### Playback procedure

We broadcasted songs with a Foxpro Fury GX7 remote-controlled autonomous amplifier, connected via a 3 m cable to a Hortus CT30 loudspeaker (frequency response: 65 Hz to 21 kHz±6 dB), at the intensity estimated to be normal for the birds (mean ± SE: 89.9±0.8 dB measured at 1 m from the loudspeaker with a Brüel & Kjaer 2235 sound level meter, linear setting). The loudspeaker was positioned on the ground at approximately 5 m from the boundary within the subject's territory, from the side shared with the left adjacent neighbour NL and from the side shared with the right adjacent neighbour NR (Fig. 3). The experimenter stood at 20 m from the loudspeaker. The playback was initiated when the subject was standing on the ground inside its territory at more than 10 m from the loudspeaker. Each subject received 2×2 playback treatments: two playbacks (S1 and S2, Fig. 3) from each side of the two opposite neighbours. Once the two playbacks from one side were completed, the loudspeaker was moved to the opposite side. The order in which stimuli were presented was balanced and presentations were separated by at least 5 min for playbacks from one side and separated by at least 10 min when we moved the loudspeaker to the opposite side.

#### Responses measured and statistical analyses

For each trial, the response of the subject was observed during 160 s, corresponding to the broadcast of 80 s of continuous song and 80 s of post-playback silence. Skylark males display a very strong territorial behaviour with stereotyped patterns, which are easy to observe [25]. We used a palm-pilot 3Com equipped with FIT program [26] to record the behavioural responses. Eleven response measurements were recorded to assess the effects of the different categories of songs broadcasted (Table 1).

Two variables (duration of movement between 10 and 5 m and between 5 and 0 m) revealed identical measurements (Pearson correlation, p = 1) with 2 other variables (respectively time spent between 10 and 5 m and between 5 and 0 m) indicating that subject were always moving and not resting whilst they spent time within 10 m of the loudspeaker. We therefore considered only duration of movements for the analysis. The skylarks gave very few calls in response to the playbacks (overall we recorded only four calls during the 40 playbacks) and we excluded this variable from further analysis. We used a principal component analysis (PCA) after examination of the correlation matrix to create composite scores reflecting the measures of responses observed during the playback experiment. These composite scores provide an estimate of the response strength with higher response strength indicating a higher level of aggressive motivation. We then compared the responses to the different stimuli using GLMs (general linear models). The dependent variables were the PCA scores, the order of stimulus presentation and the type of stimulus (S1 or S2) were the independent variables. Subject identity was included as a random factor. Wilcoxon signed-rank tests were performed to compare single response variables loading significantly on PCA scores.

## Results

### Acoustic and repertoire parameters

The ascending phase of the song flight (26.8 s±2.3) was shorter than the level phase (129.5 s±12.7). All acoustic parameters varied between the song of the two phases of flight (ascending vs. level) except for the rhythm. Values for peak frequency, quartile 25%, 50% and 75%, tempo and versatility were all higher in the song of the ascending phase than of the level phase. In contrast, bandwidth values and all temporal parameters (except tempo) were lower in the song of the ascending phase than of the level phase (Table 2).

**Table 2:** Comparison of mean values (± SEs, n = 20 subjects) of parameters for the level and the ascending phase of the flight song.

Parameters		Phase of flight song		F	p
		Ascending	Level		
Frequency (Hz)	peak frequency	4074 ± 25.7	3954 ± 30.2	14.79	**p<0.0003**
	bandwidth	1755 ± 30.5	1859 ± 36.6	5.26	**p<0.025**
	quartile 25%	3636 ± 20.5	3506 ± 25.6	18.81	**p<0.0001**
	quartile 50%	4128 ± 18.6	3986 ± 26.7	23.04	**p<0.0001**
	quartile 75%	4674 ± 17.9	4495 ± 27.6	31.94	**p<0.0001**
Temporal	duration (ms)	137.9 ± 2.2	158.9 ± 3	42.22	**p<0.0001**
	interval (ms)	186.1 ± 2.5	212.5 ± 3	57.94	**p<0.0001**
	gap (ms)	48.33 ± 0.7	54 ± 2	18.33	**p<0.0001**
	rhythm	2.85 ± 0.05	2.976 ± 0.07	2.79	**0.0999**
	tempo	5.404 ± 0.071	4.743 ± 0.08	62.58	**p<0.0001**
Versatility	SylVer	8.309 ± 0.13	6.778 ± 0.2	59.40	**p<0.0001**
	TrVer	8.005 ± 0.12	7.152 ± 0.1	30.56	**p<0.0001**
	TotVer	68.91 ± 1.7	52.03 ± 2.11	56.92	**p<0.0001**

Significant p-values are given in bold.

The parameter ‘gap duration’ was not normally distributed, even after transformation, and was therefore excluded from the pDFA analysis that requires variables to be normally distributed [22]. The pDFA showed that all syllables could be correctly attributed to the relevant phase of the flight based on the acoustic parameters (pDFA, 1000 permutations, correct classifications 68/80, p = 0.001). Results of the pDFA also showed that temporal and sequential parameters (duration, interval, tempo, SylVer, TrVer and TotVer) were variables that differed most between the song produced during the ascending and the level phase (coefficient of discriminant function ≥0.05).

### Territorial responses to the playback experiment

**Figure 4:**
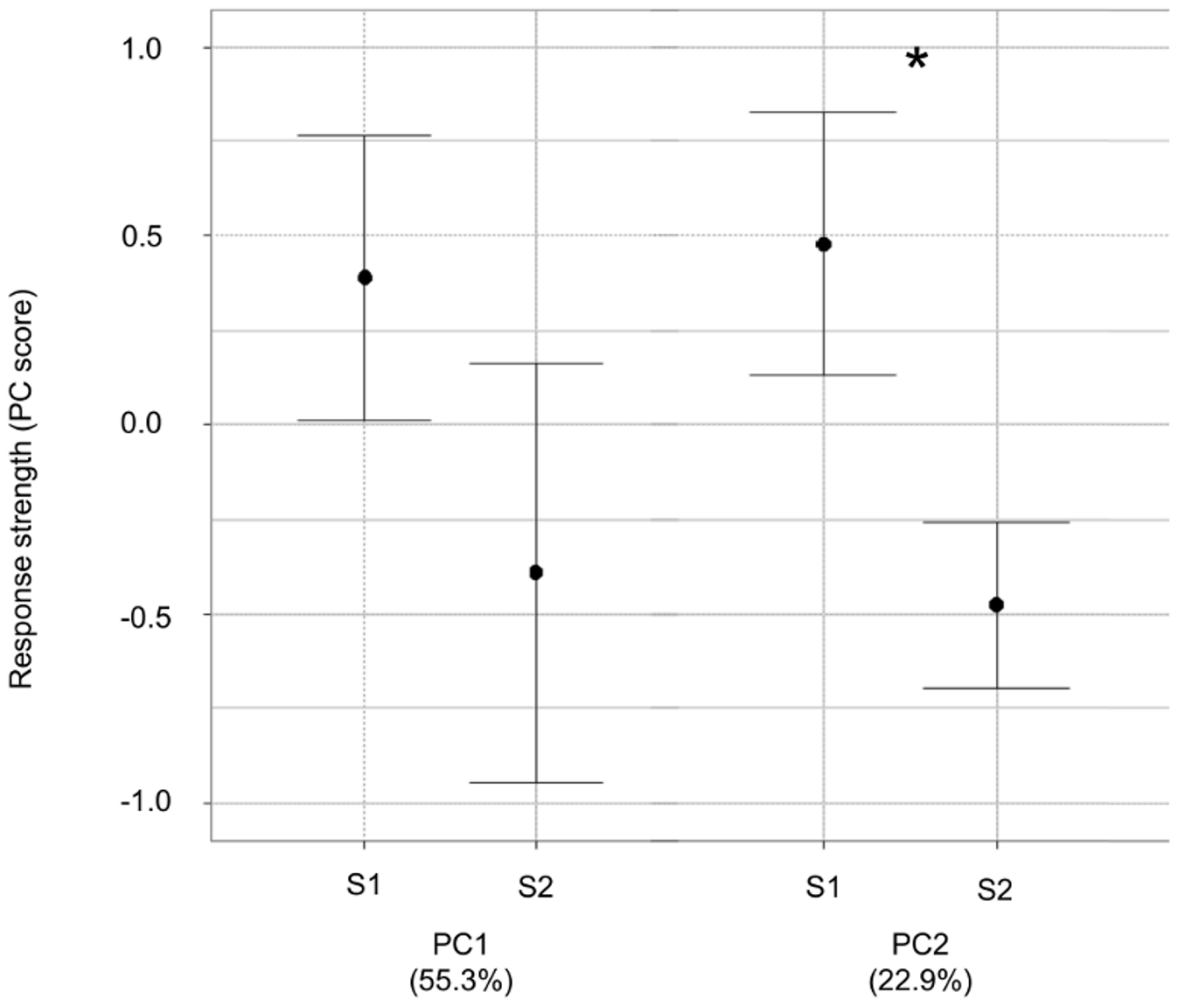
Skylarks respond more strongly to S2 stimuli. Mean scores (±SE) of the first and the second principal components for responses to the two stimuli (S1: level phase of flight song from neighbour of the opposite side; S2: ascending phase of flight song from neighbour of the opposite side). * p = 0.024. Variance explained by the scores is given in parentheses. There was no effect of the order of presentation (GLM, F = 0.53 p>0.1).

All the tested males (n = 10) reacted to at least one out of the four different stimuli presented (25 out of the 40 stimuli elicited a response). We extracted two principal component scores (PCs) with eigenvalues greater than 1.0, explaining 78.25% of the variance in the response variables (Table 3). The total duration of movements, the duration of movements between 10 and 5 m, the duration of movements between 5 and 0 m, loaded most strongly on PC1 with positive values while the latency to approach at less than 10 m, latency to approach at less than 5 m, latency before the first movement loaded negatively on PC1 (Table 3). Thus, higher PC1 scores indicated more locomotion close to the loudspeaker with a shorter latency of response and can be considered as a more aggressive response than lower PC1 scores. PC1 scores were not significantly different in response to S1 and S2 stimuli (GLM, F = 1.43, p = 0.24, Fig. 4). With regard to locomotor behaviour and latency to approach, subjects responded as strongly to one type of stimuli as to the other. Thus, they did not vary their response depending on the position of the song of the ascending and level phases in the stimuli. The PC2 explained 22.95% of the variance in the responses measured. The examination of the component loadings (Table 3) showed that the latency before the first song and the duration of songs loaded most strongly on PC2. PC2 scores were significantly different in response to S1 and S2 stimuli (GLM, F = 5.69, p = 0.024, Fig. 4). When comparing the single variables of response towards S1 and S2, we found a significant difference in the song duration (responses towards S1: 24 s±8.9, S2: 5 s±2.7; Wilcoxon matched-pairs signed-ranks test: Z = 38.5, N = 10, p = 0.044) but only a trend in the latency before the first song (response towards S1: 124.7 s±9, S2: 147 s±7.8; Wilcoxon matched-pairs signed-ranks test: Z = 8.5, N = 10, p = 0.097). Male skylarks were thus vocally more active, singing for a longer duration, when responding to stimuli in which song of the level phase of the flight was coming from the incorrect side (Fig. 4).

**Table 3:** Factor loadings of the response measures on the first (PC1) and second (PC2) principal component in playback experiment. Variance explained by the PC scores is given in parentheses.

Response measures	PC1 (55.3%)	PC2 (22.9%)
Total duration of movements	**0.4282**	0.1493
Latency before the first movement	**−0.3758**	−0.1596
Latency to approach at less than 10 m	**−0.4233**	0.1912
Latency to approach at less than 5 m	**−0.3875**	0.3437
Duration of movements between 10 and 5 m	0.3330	−0.3368
Duration of movements between 5 and 0 m	**0.3516**	−0.1638
Latency before the first song	−0.2773	**−0.5196**
Duration of songs	0.1862	**0.6224**

### Sharing of syllables and sequences of syllables between individuals in the two flight phases

The stronger vocal responses of the skylarks towards the playback stimuli, in which the song of the level phase of the flight came from the incorrect side, indicated that the individual signature might be coded rather in the song produced during the level than the ascending phase of the flight. This might be due to the song of the level phase containing certain syllables or sequences of syllables which carry the individual signature. To examine this possibility, we compared the sharing of syllables and sequences of syllables between neighbours produced during the ascending and the level phases of the flight. We predicted that if skylarks encode individuality by singing certain syllables or sequences of syllables, then neighbours should share less in the song of the level phase than in the ascending phase. In contrast to this expectation, we found that individuals shared the same amount of syllables and sequences of syllables, irrespective of the phase of flight song considered. Indeed, repertoire sharing did not differ between the two phases: pairwise comparison of males within a given locality revealed the same level of syllable sharing for ascending and level phase (mean RS ascending  = 0.10±0.03, mean RS level  = 0.16±0.03, exact paired permutation test using Monte Carlo method, n = 9, p = 0.09). Likewise, skylarks had the same level of sequence sharing in the song of the two phases (mean RS ascending  = 0.11±0.04, mean RS level = 0.13±0.04, exact paired permutation test using Monte Carlo method, n = 9, p = 0.73).

## Discussion

### The structure of the song of the skylark differs depending on the phases of flight

Skylarks exhibited variation in their song according to the different phases of their flight song. Frequency parameters (peak frequency, bandwidth, quartile 25%, quartile 50% and quartile 75%), temporal parameters (duration, interval, gap, tempo) and song versatility varied with the flight phase. The temporal parameters varied more strongly than the frequency parameters between the phases, and this was also confirmed by the discriminant function analysis.

In particular, our results indicate that the tempo of the song was faster during the ascending phase with shorter syllables and shorter inter-syllable-gaps than during the level phase. According to [27], the frequency of wing beats is higher during the ascending phase (10.7 Hz) than during the level phase (7.2 to 9.0 Hz) in skylarks. In many species, wing beat frequency is correlated with respiratory rate [28] [29]. In addition, the take-off phase in birds is assumed to be more costly in energy than the phase of level flight (e.g. [30]) and it could involve greater oxygen consumption [31]. Therefore, in skylarks a higher respiratory rate during the ascending phase could induce shorter syllables and shorter gaps and hence a faster tempo. Examining respiratory rate and oxygen consumption directly would be needed to confirm this interpretation.

Versatility, indicating song complexity, was higher during the ascending phase. This could be related to the high tempo in this first phase. Indeed, the ‘anti-exhaustion’ hypothesis [32] postulates that to maintain a high tempo, a bird should produce a more versatile song: As producing the same syllable types should weary the combination of muscles involved, to maintain a fast tempo, a bird should frequently change syllable types. Our results are consistent with this assumption. Thus, a higher versatility during the ascending phase could help skylarks to maintain a higher song tempo adapted to the higher respiratory rate which might be required by the take-off.

Frequency bandwidth, that is, the difference between syllable maximum and minimum frequency was narrower during the ascending than during the level phase of flight in skylarks. Podos [33] showed that a compromise exists between the production of fast trills and the production of trills covering a wide frequency bandwidth and that it is probably due to a trade-off between trill rates and magnitudes of possible vocal tract modulations. One might therefore speculate that in the ascending phase where constraints are probably more prominent, the birds could not produce syllables with a wider bandwidth.

### Song produced during the level phase of the flight seems to be more important for individual recognition

The second aim of this study was to examine whether the phases of the flight song might play a role in the coding strategy, that is, whether individuality might be predominantly coded in one rather than the other phase of the flight song. Song produced during the flight phase that probably requires more energy (upward flight of the ascending phase) might contain less information on individuality as birds are burdened by energetic costs. Moreover we have found that some acoustic parameters differed between the song of the ascending and the level phase and we can assume that the coding rules were not the same in these different phases. Our playback experiment suggests that the individual signature might be located in the song of the level phase of the flight. Indeed the different types of stimuli elicited significantly, although subtly, different vocal responses (PC2). Male skylarks were vocally more active when exposed to stimuli in which the song of the level phase of the flight was from the incorrect neighbour, that is, when the level phase of stimuli mimicked a displaced neighbour. However, we have to acknowledge that, independently of the stimuli tested, all the responses measured for the playback were weak. Such overall weak reactions might be due to the fact that all our stimuli contained the group signature (i.e., syllable sequences shared by the group members), as they originated all from males within the same group of the subject. Thus, subjects probably identified our stimuli as songs produced by birds from the same group and therefore exhibited a low territorial response (“dear-enemy effect”, [4]). We cannot exclude that the inability for the tested subjects to visually locate the intruder could explain why we found no differences in locomotor behaviour and latency to approach (PC1) in response to playback. We believe nevertheless that the presence of the group signature in all stimuli is a more plausible explanation of the relatively low territorial responses, as in a previous study no visual stimuli were presented neither. Still playbacks in this previous study evoked robust responses [4]. As the duration of songs played back were short compared to a natural song, we cannot rule out the possibility that discrimination might occur only after a certain period of priming, that is, after a certain duration of the ascending phase of the flight. Nevertheless, as the duration of our songs stimuli of the ascending phase were longer than the average of the natural ones, and as skylark males react very quickly when challenged by intruder songs, such a possibility seems unlikely. Individuality could be coded at the level of the frequency and temporal parameters found to be different between the song produced during the two phases, and/or by particular syllables or sequences of syllables. The fact that the amount of sharing of syllables and sequences of syllables between neighbours did not differ between the song of the ascending and the level phase of the flight suggests, however, that it is less likely that particular syllables or sequences carry the individual code. How exactly individuality is coded (intra-syllable acoustic parameters, sequential parameters or a combination of these two) remains an open question and further investigations will be needed.

A segregation of different information (group identity, individual identity) in different parts of the song might resolve coding conflicts arising from the need to carry both the individual signature (i.e. individually stereotyped song features) and the group signature (i.e. requiring that members of a group share common song features) [34]. However, our study revealed that sharing of sequences of syllables between neighbours did not differ between the ascending and the level phase which indicates that those shared sequences of syllables that act as a password for group identity are equally distributed in the two phases of the songs. Thus, while skylark males give their group signature throughout the song, they seem to signal their individual identity later during the flight. This suggests that the most important relationship to be established might be the relationship between members of a group.

In conclusion, our study suggests that the different flight phases might have an impact on the acoustic properties of skylark's song maybe due to different song production constrains. Temporal parameters and versatility turned out to be the parameters which were the most strongly influenced by the phase of the flight perhaps because of the links between flight, respiration and song production. We also found that the individuality seems to be coded rather in the level phase of the flight song. Thus, the reported difference in acoustic parameters could also be due to a segregation of coded information in the different phases of the flight.
